# United States Congressional COVID-19 Legislation: Recent Laws and Future Topics

**DOI:** 10.5811/westjem.2020.7.48891

**Published:** 2020-08-17

**Authors:** Marisa K. Dowling, Aisha T. Terry, Natalie L. Kirilichin, Jennifer S. Lee, Janice C. Blanchard

**Affiliations:** George Washington University Medical Faculty Associates, Department of Emergency Medicine, Washington, District of Columbia

## INTRODUCTION

Nothing is normal now, least of all the United States Congress. As the novel coronavirus (COVID-19) pandemic devastates Americans’ health and livelihoods, Congress has passed sweeping legislation to address the nation’s parallel medical and economic crises. These legislative interventions have important implications for emergency physicians—as frontline workers, family members, and advocates. This article summarizes the new laws’ most relevant provisions for emergency physicians.

## LEGISLATION TO DATE

To date, the US Congress has passed four coronavirus relief bills (Table 1).

### First Law

On March 6, 2020, Congress passed the first coronavirus relief law (*Coronavirus Preparedness and Response Supplemental Appropriations Act*, Public Law 116–123). At a cost of $8.3 billion, the law focuses on immediate pandemic response efforts, including funding to create viral test kits, vaccine and drug development, and aid for state and local health departments.

### Second Law

At a price tag of $192 billion, Congress enacted the *Families First Coronavirus Response Act* (P.L. 116–127) on March 18, 2020. The law provides significant aid to individuals and families suffering from the economic effects of COVID-19 related shutdowns, including expanded unemployment benefits and emergency paid sick leave for eligible workers. Of note, “frontliners” (such as emergency physicians) were excluded from sick leave expansions. This exclusion was intentional due to concerns for potential healthcare staffing shortages if sick leave expansions included essential medical workers.

### Third Law

On March 27, 2020, Congress passed the largest ($1.7 trillion) stimulus law in US history, the *Coronavirus Aid, Relief, and Economic Security (CARES) Act* (P.L. 116–136).^1^ The *CARES Act* dramatically escalated Congress’ response to the virus’ staggering economic impact through direct stimulus cash payments to most Americans, expanded unemployment benefits, and aid to businesses.

Most notably for healthcare providers, the *CARES Act* created the Provider Relief Fund (PRF) with $100 billion in aid for healthcare organizations and clinicians of all types to assist with lost revenues and COVID-19 preparedness expenses. Congress gave the Department of Health and Human Services (HHS) considerable discretion in the distribution of PRF monies. HHS subsequently faced an onslaught of funding appeals from various provider groups. To date, PRF disbursements have included the following:

- $30 billion based on a provider’s share of 2019 Medicare fee-for-service reimbursements. 320,000 providers received funds through this mechanism.
- $20 billion based on a provider’s share of net patient revenue; 15,000 providers received funds through this disbursement.
- $15 billion for providers serving high numbers of Medicaid and Children’s Health Insurance Program (CHIP) patients. Data on the number of recipients is pending.
- $13 billion for hospitals with high numbers of low-income and uninsured patients based on disproportionate share hospital (DSH) funding. Information on the number of recipients is pending.
- $22 billion for hospitals with high numbers of COVID-19 patients.
  - $12 billion was first distributed to 395 hospitals with 100 + COVID-19 patients before April 10, 2020, which averaged to $76,975 per eligible admission.
  - A second round of payments totaling $10 billion started July 20, 2020, for hospitals that had more than 161 COVID-19 admissions (ie, averaging one COVID-19 admission per day) between January 1–June 10, 2020, and/or had a high intensity of COVID-19 admissions (exceeded the average ratio of COVID-19 admissions/bed). One thousand hospitals are expected to benefit from these payments, which average out to $50,000 per eligible admission. HHS will consider hospitals’ funding from the first round when allocating the second round of payments. HHS has stated that it plans to evaluate and provide additional relief funds to future COVID-19 hotspot hospitals as monies allow.
- $11 billion for over 4000 rural hospitals (including critical access hospitals), rural health clinics, and rural health centers. Payments included a minimum base payment ($100,000 for clinics, $1 million for hospitals) plus a percent of the site’s annual expenses.
- $4.9 billion for skilled nursing facilities (SNF). So far, 13,000 SNFs have benefited from such funding.
- $500 million for the Indian Health Service.

HHS also reserved $12 billion for reimbursing providers caring for uninsured COVID-19 patients. Of note, for all the above funds, HHS requires that providers complete an online application (which includes questions about the entity’s finances) by certain deadline(s) and accept terms and conditions (which include a prohibition against balance billing COVID-19 patients).

Overall, how and whether these funds will trickle down to individual emergency physicians—many of whom have seen their hours cut or faced furlough— remains to be seen. Given that funds are largely disbursed through billing mechanisms, employed and group practice physicians will likely not receive direct payments from the relief fund. Rather, the vast majority of PRF funding has gone to hospitals or other large care organizations, rather than to individual clinicians.^2^ Solo practitioners and/or independent contractors who manage their own billing, however, can receive funds directly from the PRF via their tax identification number.

### Fourth Law

Following this whirlwind of COVID-19 related legislation, Congress entered a legislative stalemate for about a month. Ultimately when funding for small business loans lapsed, Congressional leaders compromised, and on April 24 passed the *Paycheck Protection Program and Health Care Enhancement Act* (P.L. 116–139). At a cost of $396 billion, the law limits itself to supplemental funding for small business loans and the PRF ($75 billion).

### Next Bill

Congress sank into a period of political gridlock after the fourth law’s passage. In an attempt to spur negotiations, on May 15 the House of Representatives passed the *Health and Economic Recovery Omnibus Emergency Solutions (HEROES)* Act (H.R. 6800). The bill represents House Democrats’ ideal version of the next COVID-19 relief package, which they hope will set the terms of the coming debate.

The *HEROES Act* comprises a wide range of provisions from significant aid to state and local governments to direct cash payments to most Americans. Provisions that are most likely to affect emergency physicians include the following:

- **Hazard Pay –** Calls for a “Heroes Fund” of $200 billion in “premium” pay for essential workers, such as health professionals, sanitation personnel, and grocery store employees. Workers earning less than $200,000 would be eligible to receive up to $10,000 in hazard pay. Workers earning more than $200,000 would be capped at $5,000. In order for employees to get any of this money, their employer would need to apply to the federal government for a “Heroes Fund” grant. The employer would then distribute the grant money to eligible workers in the form of a supplement to the workers’ hourly wage ($13/hour) for work done during the public health emergency (PHE) up to the worker’s maximum eligibility ($10,000 or $5,000). Workers could not apply for funds directly.
- **Personal Protective Equipment (PPE) Standards –** The Occupational Safety and Health Administration (OSHA) previously issued guidance on what qualifies as proper PPE for health care workers; this includes gloves, gowns, goggles/face shield, and National Institutes of Safety and Health-certified, disposable N-95 filter facepiece respirators or higher.^3^ The *HEROES Act* tasks OSHA with strictly enforcing these PPE standards for infection control. Moreover, the law would prohibit employers from retaliating against workers who report infection control problems and protect employees who wish to use their personally owned, more protective PPE at work, if not provided by the employer.
- **Student Loans –** Grants up to $10,000 in federal and $10,000 in private student loan forgiveness to eligible borrowers who are struggling financially. It also extends the pause on student loan payments until September 2021 for nearly all federal loan types.^4^ The *CARES Act* had already automatically paused federal student loan payments, set interest rates to 0%, and decreed that any “non-payments” through September 2020 will still qualify toward student loan forgiveness program payment obligations.
- **Provider Funding** – Adds $100 billion to the *CARES Act* PRF, bringing the total across all bills to $275 billion.

Whether these provisions become law hinges on future Senate deliberations.

## LOOKING FORWARD

While the ground is constantly shifting as Congressional negotiations proceed, most observers believe Congress will pass a bill this summer given the nation’s ongoing economic crisis and the continued rise in COVID-19 cases. Yet some commentators feel the next bill will be the last “definite” COVID-19 legislation passed before Congress succumbs to its traditional election year-related doldrums.

### Liability Reforms

While not included in the *HEROES Act*, liability reform may be central to the next COVID-19 package. Senate Majority Leader Mitch McConnell has insisted that the next coronavirus package include broad liability protections for medical professionals and businesses to stop a “second pandemic” of “lawsuits against doctors, nurses, hospitals, and brave business people who are opening up” covering the period from December 2019–December 2024.^5^,^6^ In the meantime, 26 states previously had or recently enacted some type of civil liability immunities and/or Good Samaritan protections for physicians during the public health emergency (PHE).^7^

### Telehealth

In response to the pandemic, the Centers for Medicare & Medicaid Services (CMS) significantly relaxed a number of previous telehealth regulations in order to reduce the spread of the virus and make it easier for patients to receive needed medical care. To date, CMS has waived rules regarding the following:

- *Geographic Limits* – Now patients can use telehealth anywhere in the US (urban or rural), rather than only certain qualifying rural areas.
- *Site of Care* – CMS removed “originating site” requirements. As a result, patients can now use telehealth at home, rather than having to go to certain health facilities to use the technology.
- *Privacy & Security* – Providers can now use common, unsecured, non-HIPPA compliant applications such as Zoom, Skype, and Facetime for telehealth.
- *Technology* – Audio-only phone calls, in addition to audio-visual telecommunications, now qualify as telehealth. This especially helps those unfamiliar with newer devices/technology and those who lack access to broadband Internet (such as rural areas).
- *Medical Care* – Physicians may treat nearly any condition via telehealth with no prior in-person patient-doctor relationship required. Of note for emergency medicine, permitted telehealth services include the Emergency Medical Treatment and Labor Act-required medical screening exams and tele-triage.
- *Payments –* Medicare now pays equally for in-person and telehealth visits.

Many state Medicaid programs and private insurers have similarly expanded their telehealth offerings and increased payments. As a result of these regulatory changes, telehealth usage has grown significantly nationwide. Specifically, Medicare telehealth visits jumped from 13,000 per week prior to the pandemic to 1.7 million per week by late April.^8^ Analysts predict that telehealth visits across all specialties and payers could top one billion visits this year alone. 9

However, the clock is ticking on the telehealth expansion. All of CMS’ relaxed rules and increased payments are set to expire when the HHS Secretary (in consultation with other public health experts) determines that the PHE is over. By law, PHE declarations last 90 days and can be renewed in 90-day increments as long as the HHS Secretary determines it is needed. Notably, the PHE had been set to expire on July 25, 2020, but HHS officially extended the PHE for an additional 90 days to a new end date of October 23, 2020. Ultimately, industry insiders hope that Congress will move to make a number of the new telehealth rules permanent after the PHE. Even so, many questions (reimbursement, coverage, access, security, privacy, and inter-state medical licensure) remain over how to implement telehealth services going forward.

## CONCLUSION

Emergency physicians have faced unprecedented challenges during this pandemic. As Congress attempts to mitigate the ongoing COVID-19 crisis, continued advocacy from emergency physicians is needed to ensure that the needs of our patients, communities, and profession remain prioritized. Consider reaching out to your local, state, and federal government representatives regarding your frontline experiences and the need for their support on the issues most critical to our specialty and society.

## Figures and Tables

**Table 1:** Key provisions of COVID-19 relief laws.

Law	Date	Cost	Key Healthcare Provisions	Other Notable Provisions
*Coronavirus Preparedness and Response Supplemental Appropriations Act* (Public Law 116–123)	March 6	$8.3 billion	Immediate pandemic response $6.7 billion for test kits, vaccine and drug development, and state and local health departments $100 million in grants to rural/underserved communities Health and Human Services Secretary given the authority to loosen Medicare telehealth restrictions	$20 million in small business loans $1.6 billion in international COVID-19 response aid
*Families First Coronavirus Response Act* (P.L. 116–127)	March 18	$192 billion	Free COVID-19 testing for the insured Requires all insurers to cover COVID-19 treatment, though cost-sharing requirements (co-pays, deductible, etc.) remain in effect. Increases federal matching funds (Federal Medical Assistance Percentages, FMAP) for Medicaid by 6.2%	$8 billion for nutrition assistance programs Expanded unemployment insurance benefits Emergency paid sick leave for eligible workers (“frontliners” excluded)
*Coronavirus Aid, Relief, and Economic Security (CARES) Act* (P.L. 116–136)	March 27	$1.7 trillion	*Payments* $100 billion Provider Relief Fund (PRF) to assist with pandemic response, lost revenues. $34 billion in advance Medicare payments to assist provider cash flow Delayed planned disproportionate share hospital (DSH) cuts *Testing & Supplies* $11 billion for state and local testing Funding for personal protective equipment (PPE) procurement and supply chain improvements. Requires any future COVID-19 vaccine to be free for insured patients	$349 billion for Small Business Administration’s Paycheck Protection Program (PPP) $25 billion for nutrition assistance programs Federal student loan debt assistance
*Paycheck Protection Program and Health Care Enhancement Act* (P.L. 116–139)	April 24	$396 billion	$75 billion more towards PRF $1 billion for COVID-19 testing for the uninsured	$321 billion more for the PPP
